# Comparison of 4′-[methyl-^11^C]thiothymidine (^11^C-4DST) and 3′-deoxy-3′-[^18^F]fluorothymidine (^18^F-FLT) PET/CT in human brain glioma imaging

**DOI:** 10.1186/s13550-015-0085-3

**Published:** 2015-03-05

**Authors:** Yasunori Toyota, Keisuke Miyake, Nobuyuki Kawai, Tetsuhiro Hatakeyama, Yuka Yamamoto, Jun Toyohara, Yoshihiro Nishiyama, Takashi Tamiya

**Affiliations:** Department of Neurological Surgery, Faculty of Medicine, Kagawa University, Kagawa, 761-0793 Japan; Department of Radiology, Faculty of Medicine, Kagawa University, Kagawa, 761-0793 Japan; Research Team for Neuroimaging, Tokyo Metropolitan Institute of Gerontology, Tokyo, 173-0015 Japan

**Keywords:** Glioma, Cell proliferation, FLT (fluorothymidine), 4DST (4′-thiothymidine), Positron emission tomography

## Abstract

**Background:**

3′-deoxy-3′-[^18^F]fluorothymidine (^18^F-FLT) has been used to evaluate tumor malignancy and cell proliferation in human brain gliomas. However, ^18^F-FLT has several limitations in clinical use. Recently, ^11^C-labeled thymidine analogue, 4′-[methyl-^11^C]thiothymidine (^11^C-4DST), became available as an *in vivo* cell proliferation positron emission tomography (PET) tracer. The present study was conducted to evaluate the usefulness of ^11^C-4DST PET in the diagnosis of human brain gliomas by comparing with the images of ^18^F-FLT PET.

**Methods:**

Twenty patients with primary and recurrent brain gliomas underwent ^18^F-FLT and ^11^C-4DST PET scans. The uptake values in the tumors were evaluated using the maximum standardized uptake value (SUVmax), the tumor-to-normal tissue uptake (T/N) ratio, and the tumor-to-blood uptake (T/B) ratio. These values were compared among different glioma grades. Correlation between the Ki-67 labeling index and the uptake values of ^11^C-4DST and ^18^F-FLT in the tumor was evaluated using linear regression analysis. The relationship between the individual ^18^F-FLT and ^11^C-4DST uptake values in the tumors was also examined.

**Results:**

^11^C-4DST uptake was significantly higher than that of ^18^F-FLT in the normal brain. The uptake values of ^11^C-4DST in the tumor were similar to those of ^18^F-FLT resulting in better visualization with ^18^F-FLT. No significant differences in the uptake values of ^18^F-FLT and ^11^C-4DST were noted among different glioma grades. Linear regression analysis showed a significant correlation between the Ki-67 labeling index and the T/N ratio of ^11^C-4DST (*r* = 0.50, *P* < 0.05) and ^18^F-FLT (*r* = 0.50, *P* < 0.05). Significant correlations were also found between the Ki-67 labeling index and the T/B ratio of ^11^C-4DST (*r* = 0.52, *P* < 0.05) and ^18^F-FLT (*r* = 0.55, *P* < 0.05). A highly significant correlation was observed between the individual T/N ratio of ^11^C-4DST and ^18^F-FLT in the tumor (*r* = 0.79, *P* = 0.0001).

**Conclusions:**

The present study demonstrates that ^11^C-4DST is useful for the imaging of human brain gliomas with PET. A relatively higher background uptake of ^11^C-4DST in the normal brain compared to ^18^F-FLT limits the detection of low-tracer-uptake tumors. Moreover, no superiority was found in ^11^C-4DST over ^18^F-FLT in the evaluation of cell proliferation.

## Background

**Figure 1:**
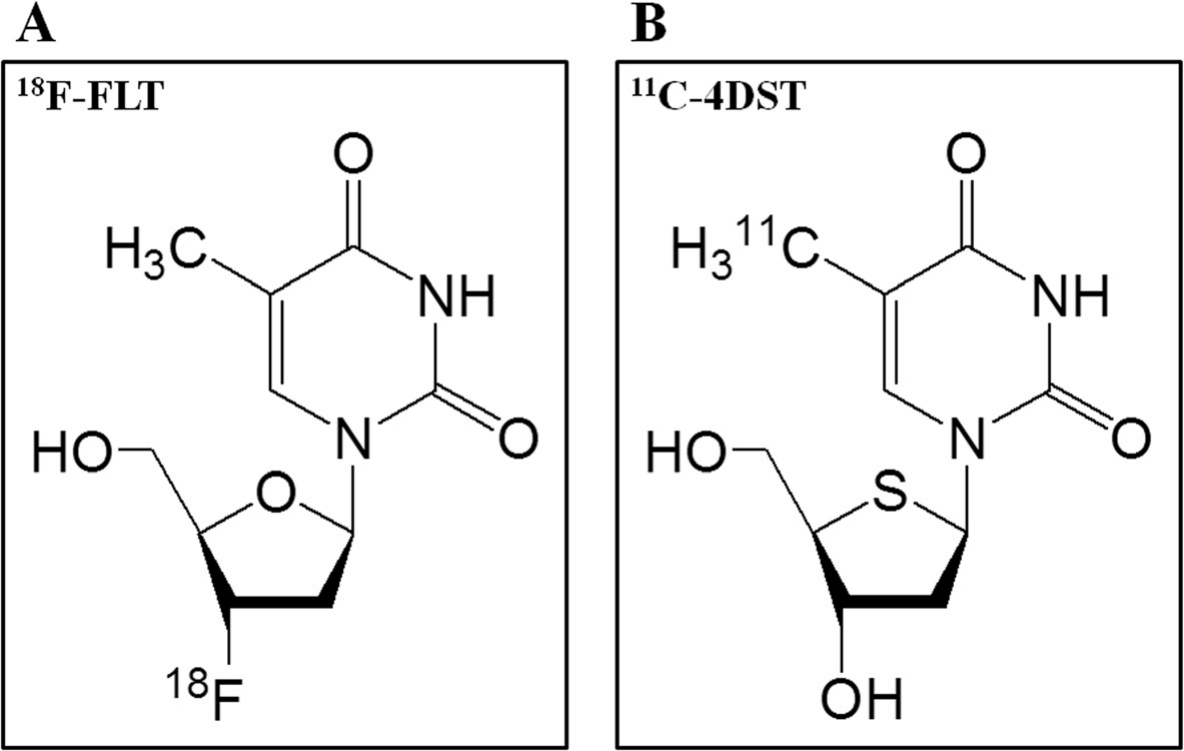
**Radiopharmaceutical structures of** ^**18**^ **F-FLT (A) and** ^**11**^ **C-4DST (B).** ^18^F-FLT, 3′-deoxy-3′-[^18^F]fluorothymidine; ^11^C-4DST, 4′-[methyl-^11^C]thiothymidine.

Imaging of cell proliferation and DNA synthesis is an attractive target for diagnosing and treating tumors. A fluorinated thymidine analogue, 3′-deoxy-3′-[^18^F]fluorothymidine (^18^F-FLT) (Figure 1A), has emerged as a promising positron emission tomography (PET) tracer for evaluating cell proliferation in various malignant brain tumors [1-4]. Fluorothymidine (FLT) is phosphorylated by thymidine kinase-1 (TK1) and trapped inside the cells. The application of ^18^F-FLT as a marker of cell proliferation is based on the assumption that cellular ^18^F-FLT trapping is a representation of thymidine incorporation into DNA [5,6]. However, previous studies including our recent report have shown that the major portion of ^18^F-FLT uptake is due to increased transport and influx through the disrupted blood-brain barrier (BBB) but not increased phosphorylation process by TK1 [7,8]. Non-enhancing tumors with an intact BBB have limited transport for the tracer, and their cell proliferation cannot be adequately assessed by ^18^F-FLT [4,9]. Moreover, little ^18^F-FLT is actually incorporated into DNA synthesis; rather, it is retained intracellularly after mono-phosphorylation by TK1 [10]. Therefore, ^18^F-FLT uptake in the tumor does not reflect the whole DNA synthesis.

Recently, Toyohara et al. developed a new thymidine analogue, 4′-[methyl-^11^C]thiothymidine (^11^C-4DST) (Figure 1B), for cell proliferation PET-imaging tracer [11,12]. ^11^C-4DST is resistant to degradation by thymidine phosphorylase and incorporated into DNA synthesis [12]. ^11^C-4DST showed high-tumor uptake (sensitivity) and high-tumor selectivity in a rodent tumor model [13]. In a pilot study in human brain tumors, ^11^C-4DST showed little uptake in normal brain tissue, resulting in low-background activity for imaging brain tumors [14]. ^11^C-4DST PET demonstrated rapid uptake in aggressive tumor masses, whereas no uptake of ^11^C-4DST was seen in clinically stable disease [14]. ^11^C-4DST might be superior to ^18^F-FLT in evaluating cell proliferation and treatment response and predicting prognosis as ^11^C-4DST uptake represents the whole DNA synthesis process. The difference of radiopharmaceutical structure between ^11^C-4DST and ^18^F-FLT (Figure 1A,B) is small; however, it has been recognized that small molecular changes of the chemical structure sometimes make greater difference in the pharmacokinetics of PET tracers. The differences of thymidine kinase selectivity, nucleoside transporter subtype, and rate-limiting step enzyme between ^11^C-4DST and ^18^F-FLT may change the characterization of PET findings [15]. No comparison has been reported between ^11^C-4DST and ^18^F-FLT for *in vivo* imaging tool of tumors. In the present study, we demonstrate our initial experience of ^11^C-4DST PET imaging and compare the images of ^11^C-4DST and ^18^F-FLT to evaluate usefulness in the diagnosis of human brain gliomas.

## Methods

### Subjects

Twenty primary (*n* = 9) and recurrent (*n* = 11) human brain gliomas (9 men and 11 women; mean age, 52.9 ± 18.6 years; range, 22 to 79 years; median, 47 years) were included in this study (Table 1). Histopathology was examined on tissue specimens obtained by biopsy (*n* = 3) or resection (*n* = 17). Twelve patients had grade IV glioma (10 glioblastoma, 2 glioblastoma with oligodendroglioma component), 6 had grade III glioma (4 anaplastic astrocytoma, 2 anaplastic oligoastrocytoma), and 2 had grade II diffuse astrocytoma. The cellular proliferation activity of the tumor was determined by measuring the Ki-67 labeling index obtained by immunohistochemical staining with anti-Ki-67 antibody (1:50, Dako, Glostrup, Denmark). The percentage of tumor cells that stained positively for Ki-67 antigen was measured in the area containing the largest number of positive cells and was regarded as representative of the tumor proliferative activity. PET studies with ^11^C-4DST and ^18^F-FLT were performed within 1 week before the surgery. The two PET studies could be performed on the same day; however, we performed the examinations on two different days to decrease a physical burden on the patients because some of the patients were ill and the mean interval between the two examinations was 2.9 ± 1.7 days (median, 2 days). All patients underwent routine magnetic resonance imaging (MRI) examination including contrast-enhanced T1-weighted MRI. The use of ^11^C-4DST and ^18^F-FLT as a PET tracer was approved by Kagawa University, Faculty of Medicine Human Subjects Ethical Committees and informed consent was obtained from all patients before PET examination.

**Table 1:** **Patient characteristics and PET data**

**Case no.**	**Age (years)/sex**	**Tumor type** ^**a**^	**Primary/recurrence**	**Ki-67 index**	**4DST**		**FLT**	
					**SUVmax**	**T/N ratio**	**SUVmax**	**T/N ratio**
1	64/F	DA	Primary	1	0.53	1.71	0.57	2.71
2	22/M	DA	Primary	5	0.53	1.51	0.28	1.75
3	46/M	AA	Primary	15	0.85	2.24	0.95	5.59
4	73/M	AA	Primary	20	2.35	6.91	2.41	14.18
5	64/M	AOA	Primary	85	1.13	3.77	1.06	7.57
6	69/F	GBM	Primary	20	1.63	4.66	1.34	6.70
7	39/F	GBM	Primary	70	1.44	4.80	1.72	7.82
8	76/F	GBM	Primary	90	6.95	18.29	3.23	17.95
9	72/F	GBM-O	Primary	70	1.57	4.76	1.35	7.11
10	34/M	AA	Recurrence	22	2.24	9.33	1.88	14.46
11	73/M	AOA	Recurrence	80	2.67	8.34	4.68	20.35
12	47/F	AOA	Recurrence	35	1.52	4.34	1.78	7.74
13	52/M	GBM	Recurrence	15	2.80	6.09	2.56	15.06
14	46/M	GBM	Recurrence	35	3.50	10.94	3.52	15.30
15	29/F	GBM	Recurrence	50	1.56	6.50	1.52	13.82
16	46/M	GBM	Recurrence	70	3.11	8.64	3.12	18.35
17	29/F	GBM	Recurrence	60	1.22	4.69	1.95	13.93
16	79/F	GBM	Recurrence	40	1.90	4.63	1.94	7.46
19	69/M	GBM	Recurrence	5	1.52	4.47	1.45	8.06
20	29/F	GBM-O	Recurrence	20	1.10	2.50	1.38	5.11

^a^DA: diffuse astrocytoma, AA: anaplastic astrocytoma, AOA: anaplastic oligoastrocytoma, GBM: glioblastoma, GBM-O: glioblastoma with oligodendroglioma component. 4DST: 4′-thiothymidine, FLT: fluorothymidine, SUVmax: maximum standardized uptake value, T/N ratio: tumor-to-normal tissue uptake ratio.

### ^11^C-4DST and ^18^F-FLT synthesis and PET acquisition

^11^C-4DST was synthesized according to the method described by Toyohara et al. [14], and the radiochemical purity of ^11^C-4DST was >95%. ^18^F-FLT was synthesized according to the method described by Martin et al. [16], and the radiochemical purity of ^18^F-FLT was >95%. All chemical reagents for tracer synthesis were purchased from commercial sources. PET examination was performed using a Biograph mCT64 PET/CT scanner (Siemens/CTI, Knoxville, TN, USA). The image systems enabled simultaneous acquisition of 74 transverses per field of view (FOV), with an intersection spacing of 3 mm, for a total axial FOV of 21.6 cm. The in-plane transverse-reconstructed resolution was 4.3 mm full width at half maximum (FWHM) in the brain FOV. No special dietary instructions were given to the patients before PET examination. Images were acquired with patients in the supine position, resting, with their eyes closed. CT data were acquired first (tube rotation time 0.6 s per revolution, 120 kV, 192 mAs, reconstructed slice thickness of 3 mm) and used for attenuation correction and anatomical localization of the tumors. For the ^11^C-4DST study, a dose of 229 to 587 MBq (mean, 391 ± 108 MBq) of ^11^C-4DST was injected intravenously, and regional emission images were obtained for 15 min, beginning 15 min after the ^11^C-4DST administration. For the ^18^F-FLT study, a dose of 237 to 342 MBq (mean, 309 ± 24 MBq) of ^18^F-FLT was injected intravenously, and regional emission images were obtained for 15 min, beginning 60 min after the ^18^F-FLT administration. Image reconstruction was performed using ordered subset expectation maximization (OSEM) with time of flight (TOF) and point spread function (PSF). The reconstruction parameters were 2 iterations and 21 subsets. The FWHM of the Gaussian filter was 3 mm.

### Data analysis

^11^C-4DST and ^18^F-FLT uptakes were semiquantitatively assessed by evaluating the standardized uptake value (SUV). A region of interest (ROI) was set manually by an observer (N.K.) around the hottest area of each lesion or the area of tumor biopsy. The maximum value of SUV (SUVmax) of the ROI was regarded as the representative value of each tumor. To calculate the tumor-to-normal tissue uptake (T/N) ratio and the tumor-to-blood uptake (T/B) ratio, ROIs were set on the normal brain parenchyma (usually contralateral normal cerebral tissue excluding ventricles) and the vertical portion of the superior sagittal sinus with the aid of PET/CT fusion images. The mean values of SUV (SUVmean) of the ROIs were calculated. The T/N and T/B ratios were determined by dividing the SUVmax of the tumor with the SUVmean of the normal brain and the superior sagittal sinus.

### Statistical analysis

All parametric data were expressed as mean ± SD. Paired Student *t*-test was used to compare the individual uptake values of ^11^C-4DST and ^18^F-FLT in the normal brain and tumor. Differences in the uptake values of ^11^C-4DST and ^18^F-FLT among different glioma grades were compared using analysis of variance. Linear regression analysis was used to evaluate the relationship between the uptake values of ^11^C-4DST and ^18^F-FLT and the Ki-67 labeling index of the tumor. The relationship between the individual ^11^C-4DST and ^18^F-FLT uptake in the tumor was also examined by linear regression analysis. Differences were considered statistically significant at a *P* value of less than 0.05.

## Results

### Visual assessment

**Figure 2:**
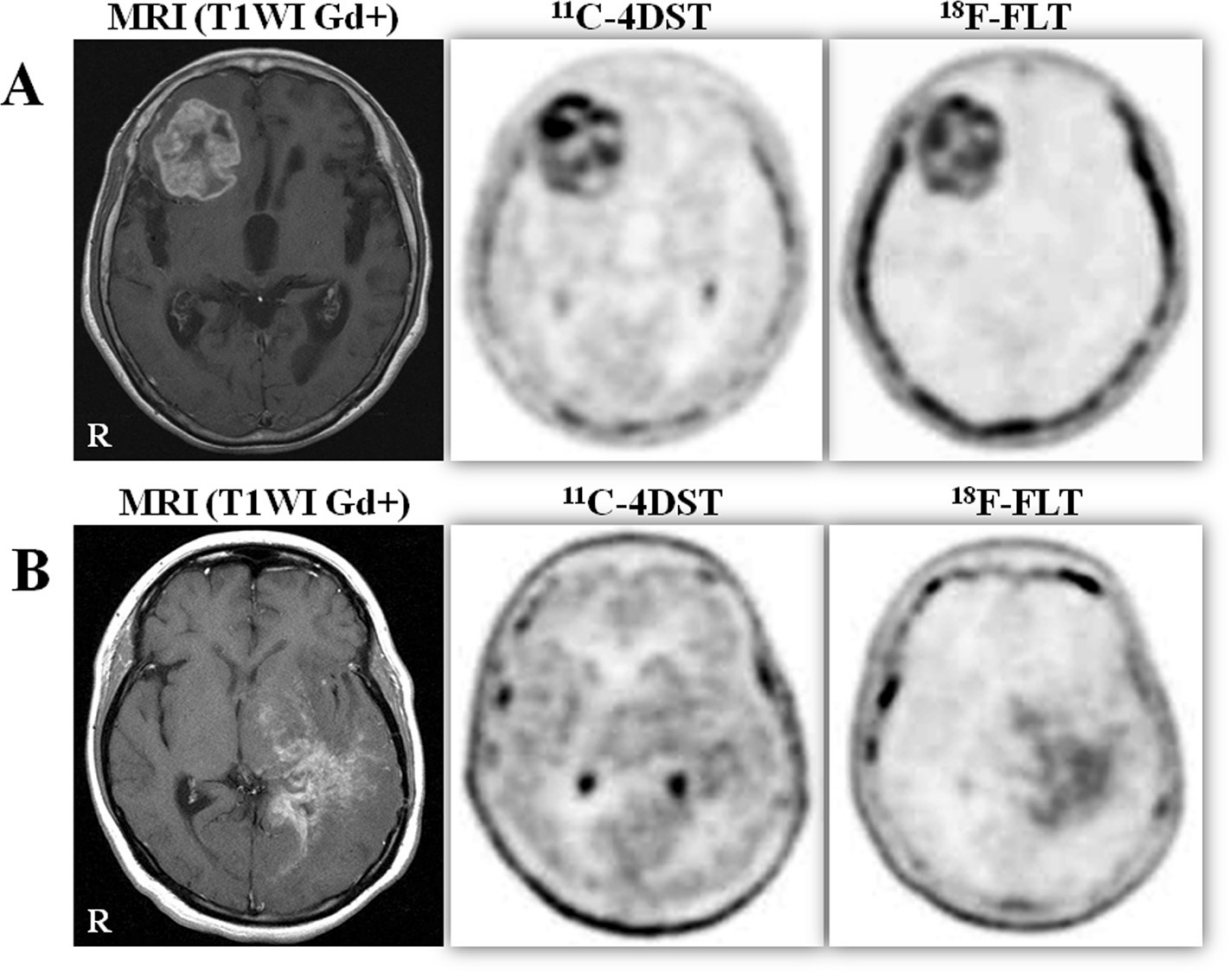
**Gd-enhanced T1-weighted MRI,** ^**11**^ **C-4DST PET, and** ^**18**^ **F-FLT PET of two patients. (A)** A 76-year-old woman with newly diagnosed glioblastoma (Case 8). Gd-enhanced T1-weighted MR image demonstrates a round lesion with irregular ring enhancement in the right frontal lobe. PET studies with ^11^C-4DST and ^18^F-FLT PET show almost identical tracer uptake in the tumor. Background uptake of ^11^C-4DST is higher than ^18^F-FLT PET. **(B)** A 29-year-old woman with recurrent glioblastoma with oligodendroglioma component (Case 20). Gd-enhanced T1-weighted MR image demonstrates multiple spotty enhancements in the right temporo-occipital lobes extending to the basal ganglia. The tumor is faintly visualized with ^11^C-4DST but well visualized with ^18^F-FLT. ^18^F-FLT, 3′-deoxy-3′-[^18^F]fluorothymidine; ^11^C-4DST, 4′-[methyl-^11^C]thiothymidine.

Both ^11^C-4DST and ^18^F-FLT showed little uptake in the normal brain, and ^11^C-4DST uptake was visually higher than ^18^F-FLT in almost all cases. ^11^C-4DST showed a high uptake in the choroid plexus, whereas ^18^F-FLT showed a faint uptake. ^11^C-4DST and ^18^F-FLT provided identical PET images of the tumor in many cases (Figure 2A). Two primary diffuse astrocytomas that had no contrast enhancement in the tumor could not be visualized with ^11^C-4DST. One of the two diffuse astrocytomas could be visualized with ^18^F-FLT but the other could not. One recurrent glioblastoma with oligodendroglioma component that had multiple spotty enhancements in contrast-enhanced MRI was faintly visualized with ^11^C-4DST but well visualized with ^18^F-FLT (Figure 2B). In total, 2 of 20 tumors were well visualized only in ^18^F-FLT PET.

### Semiquantitative analysis

In the normal brain, ^11^C-4DST uptake was significantly higher than ^18^F-FLT (SUVmean; 0.34 ± 0.06 vs. 0.19 ± 0.04, *P* < 0.001 by paired *t*-test). Individual ^11^C-4DST SUVmax in the tumor was almost comparable to ^18^F-FLT except a few cases. Therefore, the average T/N ratio of ^18^F-FLT in the tumor was significantly higher than that of ^11^C-4DST (10.55 ± 5.45 vs. 5.96 ± 3.86, *P* < 0.001 by paired *t*-test) resulting in better tumor visualization with ^18^F-FLT. The average ^11^C-4DST T/N ratio in WHO grade II (*n* = 2), III (*n* = 6), and IV (*n* = 12) gliomas was 1.16 ± 0.14, 5.82 ± 2.8, and 6.75 ± 4.25, respectively. No significant differences of ^11^C-4DST T/N ratio were observed among different glioma grades. The average ^18^F-FLT T/N ratio in WHO grade II, III, and IV gliomas was 2.23 ± 0.68, 11.65 ± 5.63, and 11.39 ± 4.78, respectively. Again, no significant differences of ^18^F-FLT T/N ratio were observed among different glioma grades.

### Correlation between Ki-67 index and uptake values of ^11^C-4DST and ^18^F-FLT

**Figure 3:**
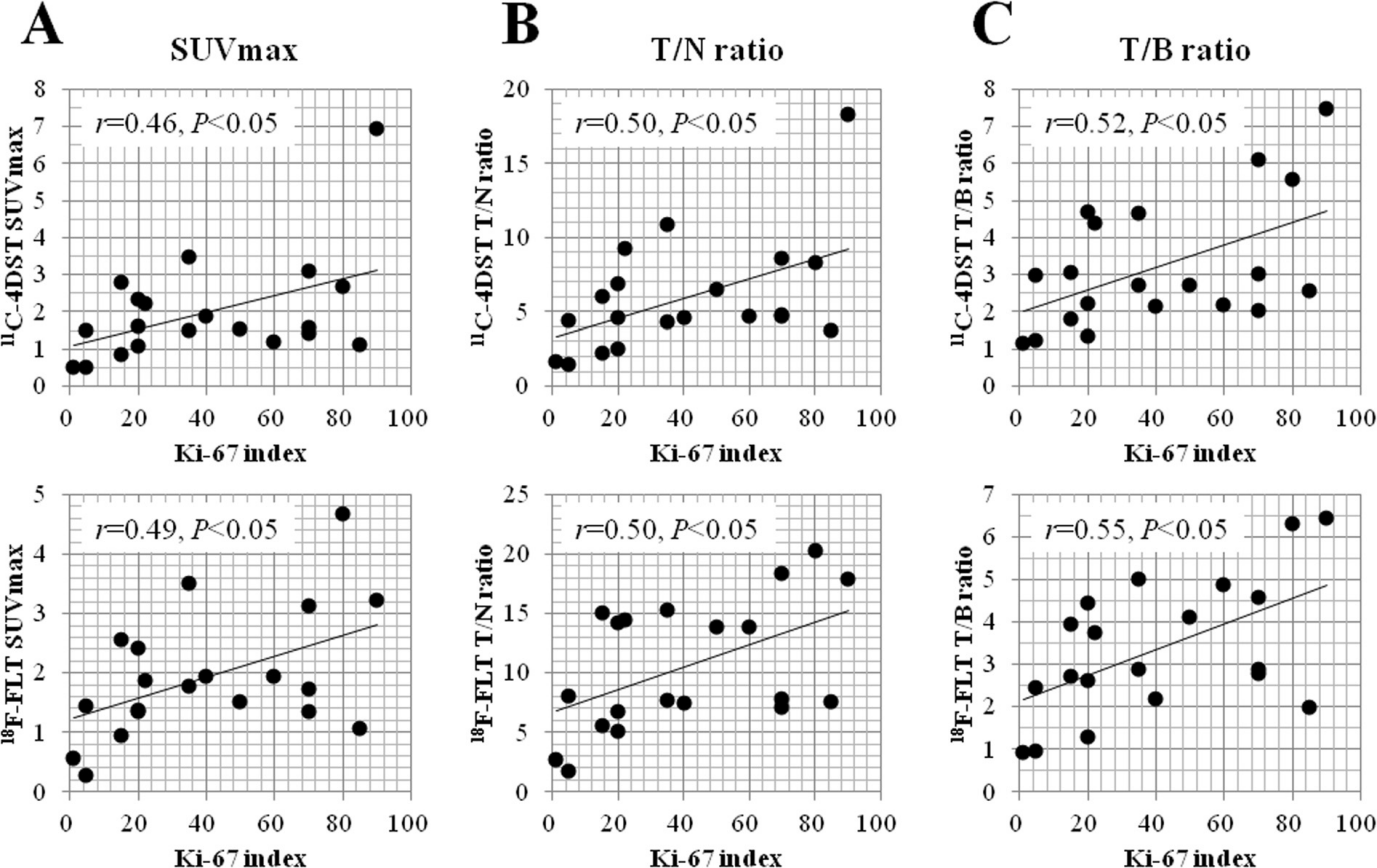
**Linear regression analysis between the Ki-67 labeling index and the uptake values of** ^**11**^ **C-4DST and** ^**18**^ **F-FLT. (A)** A significant correlation is observed between the Ki-67 labeling index in the tumor and the SUVmax of ^11^C-4DST (*r* = 0.46, *P* < 0.05) and ^18^F-FLT (*r* = 0.49, *P* < 0.05). **(B)** A significant correlation is also observed between the Ki-67 labeling index in the tumor and the T/N ratio of ^11^C-4DST (*r* = 0.50, *P* < 0.05) and ^18^F-FLT (*r* = 0.50, *P* < 0.05). **(C)** A significant correlation is also observed between the Ki-67 labeling index in the tumor and the T/B ratio of ^11^C-4DST (*r* = 0.52, *P* < 0.05) and ^18^F-FLT (*r* = 0.55, *P* < 0.05). ^18^F-FLT, 3′-deoxy-3′-[^18^F]fluorothymidine; ^11^C-4DST, 4′-[methyl-^11^C]thiothymidine; SUVmax, maximum standardized uptake value; T/N ratio, tumor-to-normal tissue uptake ratio; T/B ratio, tumor-to-blood uptake ratio.

Linear regression analysis showed a significant correlation between the Ki-67 labeling index in the tumor and the SUVmax of ^11^C-4DST (*r* = 0.46, *P* < 0.05) and ^18^F-FLT (*r* = 0.49, *P* < 0.05) (Figure 3A). Moreover, a significant correlation was observed between the Ki-67 labeling index in the tumor and the T/N ratio of ^11^C-4DST (*r* = 0.50, *P* < 0.05) and ^18^F-FLT (*r* = 0.50, *P* < 0.05) (Figure 3B). A significant correlation was also observed between the Ki-67 labeling index and the T/B ratio of ^11^C-4DST (*r* = 0.52, *P* < 0.05) and ^18^F-FLT (*r* = 0.55, *P* < 0.05) (Figure 3C).

### Correlation between individual ^11^C-4DST and ^18^F-FLT uptake values

**Figure 4:**
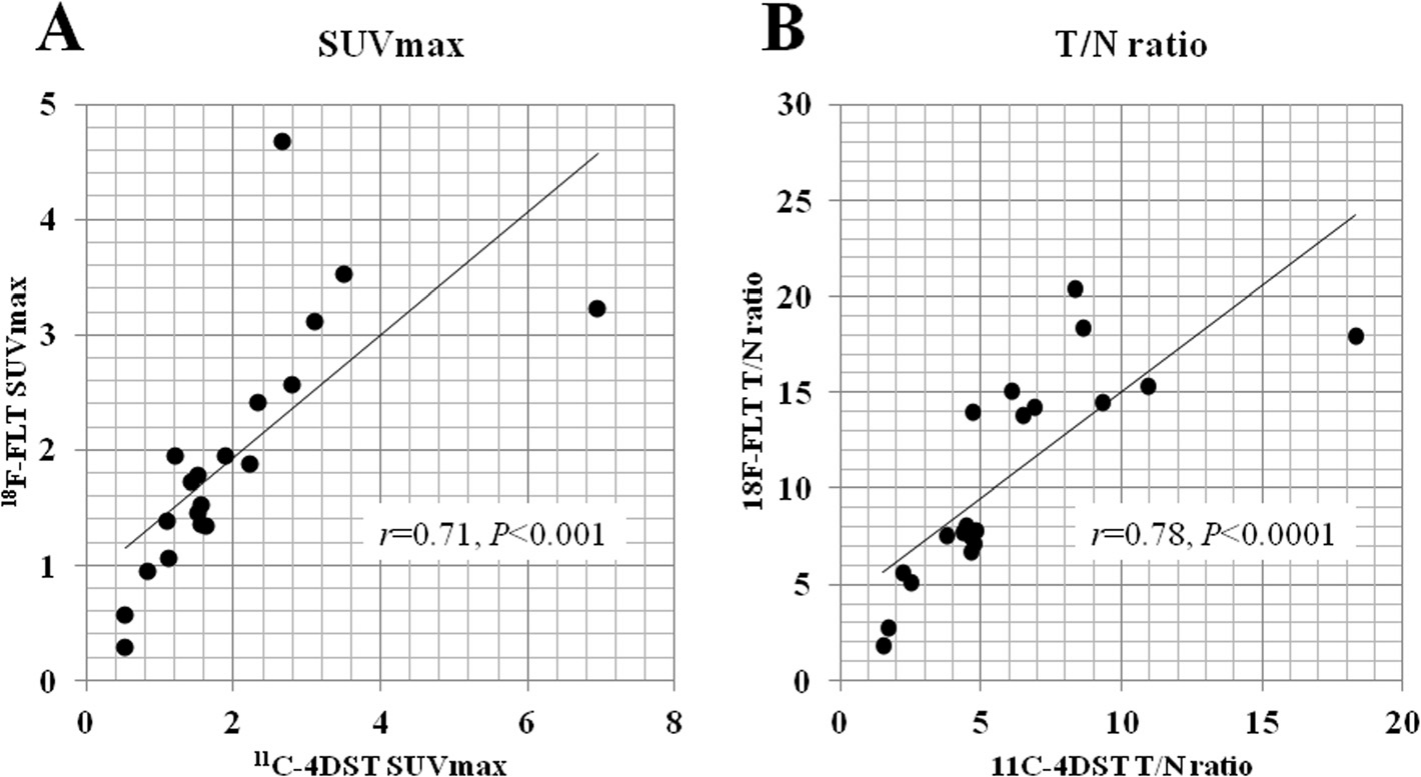
**Linear regression analysis of the individual SUVmax (A) and T/N ratio (B) of the tumor between** ^**11**^ **C-4DST and** ^**18**^ **F-FLT.** A highly significant correlation is observed between the individual SUV max of ^11^C-4DST and ^18^F-FLT (*r* = 0.71, *P* < 0.001) and T/N ratio of ^11^C-4DST and ^18^F-FLT (*r* = 0.79, *P* < 0.0001). ^18^F-FLT, 3′-deoxy-3′-[^18^F]fluorothymidine; ^11^C-4DST, 4′-[methyl-^11^C]thiothymidine; SUVmax, maximum standardized uptake value; T/N ratio, tumor-to-normal tissue uptake ratio.

A highly significant correlation was observed between the individual SUVmax of ^11^C-4DST and ^18^F-FLT in the tumor (*r* = 0.71, *P* < 0.001) (Figure 4A). A highly significant correlation was also observed between the individual T/N ratio of ^11^C-4DST and ^18^F-FLT in the tumor (*r* = 0.79, *P* = 0.0001) (Figure 4B).

## Discussion

In the present study, we demonstrate three important findings in the comparison of imaging between ^11^C-4DST and ^18^F-FLT for clinical application. Firstly, both tracers had low-background activity in the normal brain and were able to visualize the tumor well except one non-enhancing primary diffuse astrocytoma in which both tracers failed to visualize the tumor. Both tracers had similar uptake values in the tumor, but the ^18^F-FLT uptake in the normal brain was relatively lower than that of ^11^C-4DST, resulting in better tumor visualization with ^18^F-FLT. Secondly, a significant correlation was observed between the Ki-67 labeling index and the uptake values of ^11^C-4DST and ^18^F-FLT in the tumor. We hypothesized that ^11^C-4DST is superior to ^18^F-FLT in evaluating cell proliferation as ^11^C-4DST uptake represents the whole DNA synthesis process. However, the correlation coefficients of ^11^C-4DST uptake values to the Ki-67 labeling index were slightly lower than those of ^18^F-FLT. Finally, a strong correlation was observed between the individual ^11^C-4DST and ^18^ F-FLT uptake values in the tumor.

For oncological use, measurement of the cell proliferation and DNA synthesis is an attractive target for imaging. A fluorinated thymidine analogue, ^18^F-FLT, has emerged as a PET tracer for evaluating tumor-proliferating activity in various brain tumors [1-4]. However, ^18^F-FLT has several limitations in clinical use that have been reported in previous studies [4,7-9]. Most critically, little ^18^F-FLT is actually incorporated into DNA synthesis, and ^18^F-FLT uptake in the tumor does not reflect the whole of DNA synthesis [7,8,10]. With the drawbacks of ^18^F-FLT, efforts were made to produce a more attractive PET tracer that can be used to evaluate tumor malignancy and cell proliferation accurately. Toyohara et al. focused on the 4′-thiothymidine (4DST) because of its metabolic stability and close similar structure to native thymidine [11]. They synthesized ^14^C-labeled 4DST (^14^C-4DST) as a model compound of ^11^C-labeled alternative and demonstrated the evidence that ^11^C-4DST matches the concept of the ideal DNA-synthesis-imaging agent [11]. Feasibility studies of ^11^C-4DST in rodent tumor models showed higher uptake than that of ^18^F-FLT and reflect the DNA synthesis rate [12,13]. Usefulness of ^11^C-4DST for the imaging of human brain tumors with PET was investigated in a recent pilot study by comparing the images of ^11^C-4DST and [^11^C]methionine (^11^C-MET) in six patients with various brain tumors [14]. There was little uptake of ^11^C-4DST in the normal brain and rapidly growing brain tumors that were well visualized in contrast-enhanced MRI and ^11^C-MET PET were clearly seen in ^11^C-4DST PET. Although ^11^C-MET detected all the enhanced lesions in MRI, clinically stable (non-aggressive) tumors with enhancement were not detected with ^11^C-4DST. In addition, the distribution pattern of ^11^C-4DST in the tumor was not always identical to that of ^11^C-MET. Here, we report the first clinical study of comparing two nucleoside PET tracers for DNA synthesis, ^11^C-4DST and ^18^F-FLT, for *in vivo* imaging of human brain gliomas. ^11^C-4DST PET images were acquired for 15 min, beginning 15 min after the administration. The start time for imaging ^11^C-4DST was earlier than that for ^18^F-FLT PET imaging (60 min). We confirmed that ^11^C-4DST uptake and distribution in the tumor was almost fixed 15 min after ^11^C-4DST administration in a preliminary dynamic acquisition study. Two non-enhanced diffuse astrocytomas could not be visualized with ^11^C-4DST suggesting that ^11^C-4DST does not readily cross the intact BBB, and ^11^C-4DST uptake in the tumor rather depends on the influx through the disrupted BBB similar to ^18^F-FLT. The distribution pattern and uptake value of ^11^C-4DST in the tumor are almost identical to those of ^18^F-FLT except one non-enhanced primary diffuse astrocytoma and one recurrent glioblastoma with oligodendroglioma component in which only ^18^F-FLT could detect the tumor well, whereas ^11^C-4DST showed no and a faint uptake in the tumors, respectively. ^11^C-4DST uptake in the normal brain was visually and semiquantitatively higher than ^18^F-FLT. A previous clinical study reported that ^11^C-4DST is mainly metabolized by glucuronidation in the human body and largely accumulated in the liver [14]. Other metabolites could be nonspecifically accumulated in the normal brain. Furthermore, a recent study has demonstrated that 4DST but not FLT can be transported via the nucleoside transporters [15]. The active transport may cause higher uptake of ^11^C-4DST than ^18^F-FLT through the intact BBB in the normal brain though the amount of ^11^C-4DST transport is small.

In the present study, we examined 20 patients with various grades of gliomas and a mixture of primary and recurrent cases. There are critical limitations in the present study. Firstly, the total number of gliomas enrolled in this study was small for evaluating the usefulness of this new tracer, especially low-grade glioma. Secondary, primary, and recurrent cases should be separated when evaluating the usefulness of a BBB-dependent PET tracer, such as ^11^C-4DST and ^18^F-FLT. Recently, our research group has demonstrated that ^18^F-FLT PET is less useful for evaluating tumor malignancy and cell proliferation in recurrent gliomas compared with newly diagnosed gliomas [17]. In the present study, no significant differences in the T/N ratio of ^18^F-FLT were noted among different glioma grades. This finding is not in accordance with the findings of our previous [4] and recent [17] studies in newly diagnosed gliomas. Moreover, there is a significant correlation between the ^18^F-FLT uptake and the Ki-67 labeling index, but the correlation coefficient (*r* = 0.50) was lower compared with the findings of our previous (*r* = 0.89) [4] and recent (*r* = 0.81) [17] studies in newly diagnosed gliomas. These discrepancies might be due to the small number of patients and mixture of primary and recurrent cases. Radiotherapy used as an adjuvant treatment for malignant gliomas may cause loosening of the endothelial tight junctions, vascular leakage, or endothelial cell death and thus can increase vascular permeability not only in the BBB but also in the blood-tumor barrier [18]. Besides increased cell proliferation in recurrent tumors, treatment-induced breakdown of the BBB and blood-tumor barrier might contribute to the degree of ^18^F-FLT in the tumor. This may also be the case in ^11^C-4DST.

Although the short physical half-life of ^11^C is a significant limitation for routine clinical use, ^11^C-4DST has benefits with regard to lower radiation burden and diagnosis using multiple tracers in 1 day. An alternative thymidine analogue labeled with a longer half-life isotope that can be incorporated into DNA synthesis is applied`easibly for clinical use and might provide additional and alternative information. As ^11^C-4DST uptake represents the whole DNA synthesis process, ^11^C-4DST PET might be superior for evaluating treatment response and predicting prognosis compared to other tracers in patients with brain gliomas.

## Conclusions

The uptake pattern and uptake value of ^11^C-4DST in the tumor are similar to those of ^18^F-FLT, and both nucleoside tracers might be BBB dependent. Although no superiority was found in ^11^C-4DST over ^18^F-FLT in the detection of tumors and in the evaluation of cell proliferation, we consider that ^11^C-4DST is useful for the imaging of human brain gliomas with PET. Further studies are necessary to examine the usefulness of ^11^C-4DST for clinical application in evaluating treatment response and predicting prognosis.
